# Improved Production Process for Native Outer Membrane Vesicle Vaccine against *Neisseria meningitidis*

**DOI:** 10.1371/journal.pone.0065157

**Published:** 2013-05-31

**Authors:** Bas van de Waterbeemd, Gijsbert Zomer, Patricia Kaaijk, Nicole Ruiterkamp, René H. Wijffels, Germie P. J. M. van den Dobbelsteen, Leo A. van der Pol

**Affiliations:** 1 National Institute for Public Health and the Environment (RIVM), Vaccinology, Bilthoven, The Netherlands; 2 Bilthoven Biologicals, Quality Control, Bilthoven, The Netherlands; 3 Wageningen University, Bioprocess Engineering, Wageningen, The Netherlands; Health Protection Agency, United Kingdom

## Abstract

An improved detergent-free process has been developed to produce vaccine based on native outer membrane vesicles (NOMV) against *Neisseria meningitidis* serogroup B. Performance was evaluated with the NonaMen vaccine concept, which provides broad coverage based on nine distinct PorA antigens. Scalable aseptic equipment was implemented, replacing undesirable steps like ultracentrifugation, inactivation with phenol, and the use of preservatives. The resulting process is more consistent and gives a higher yield than published reference processes, enabling NOMV production at commercial scale. Product quality met preliminary specifications for 9 consecutive batches, and an ongoing study confirmed real-time stability up to 12 months after production. As the NOMV had low endotoxic activity and induced high bactericidal titres in mice, they are expected to be safe and effective in humans. The production process is not limited to NonaMen and may be applicable for other *N. meningitidis* serogroups and other gram-negative pathogens. The current results therefore facilitate the late-stage development and clinical evaluation of NOMV vaccines.

## Introduction

*Neisseria meningitidis* is a human pathogen that causes acute meningitis and septicemia with high fatality rates [1]. Outbreaks of serogroup B meningococcal disease in Norway, New Zealand, and Cuba were successfully controlled with outer membrane vesicle (OMV) vaccines, which was a milestone for the application of OMV in vaccinology [2], [3], [4], [5]. The outer membrane porin A protein (PorA) is the dominant protective antigen but varies among strains [6], [7]. Recombinant strains with multiple PorA subtypes were developed to increase vaccine coverage, resulting in a nonavalent OMV vaccine (NonaMen concept) with potential coverage of 80% [8], [9], [10], [11]. Coverage has also been increased by complementing multiple PorAs with other antigens, like fHbp or FetA [12], [13], [14]. In addition OMV improved the immunogenicity of a vaccine with purified fHbp, NadA and NHBA protein, which has been submitted for regulatory review in Europe [15], [16], [17]. The use of OMV-based vaccines therefore remains a promising approach for the control of serogroup B meningococcal disease [18].

The vaccine strains used in Norway, New Zealand, and Cuba contained lipopolysaccharide (LPS) with strong endotoxic activity. To remove most of the LPS, the production process required extraction by deoxycholate, a detergent of animal origin [19], [20], [21]. The resulting detergent-extracted OMV (DOMV) tended to aggregate, and the deoxycholate was not fully removed during purification [20], [21], [22]. These issues, together with a high strain specificity of the immune response, have delayed the late-phase development of broadly protective DOMV vaccines. Van der Ley *et al.* attenuated LPS by introducing the *lpxL1* mutation rather than physically removing it with detergent [23]. As *lpxL1*-LPS has low endotoxic activity, it enables the development vaccines based on native OMV (NOMV) for use in humans [24]. NOMV are very similar to the natural vesicles released during infection [25]. Vaccines based on NOMV therefore have several advantages over those based on DOMV, including absence of aggregation and a broader immunogenicity provided by protective lipoproteins like fHbp [22], [26], [27], [28].

NOMV vaccines against *N. meningitidis* gave promising results in preclinical and clinical studies, but were either produced at small-scale or required process steps that are undesirable at commercial scale [22], [29], [30], [31], [32], [33]. These steps include the use of growth medium with undefined components of animal origin (casamino acids), inactivation of bacteria with hazardous chemicals like phenol, or the use of excessive mechanical force to release or homogenize NOMV (e.g., blender, micro fluidizer). Other problems involve use of preservatives with toxic components (like thiomersal) and use of ultracentrifugation, which limits production scale and complicates handling. NOMV production for phase III clinical trials and commercial use demands an efficient and reliable process that fully meets present-day regulatory requirements. As such a process was unavailable for NOMV vaccines, a novel process was developed. Performance was assessed with the NonaMen concept, a vaccine with broad coverage based on nonavalent PorA. Our results show that this new process provides a robust production platform for the late-stage development and clinical evaluation of NOMV vaccines.

## Results

### Process outline and specifications

**Figure 1:**
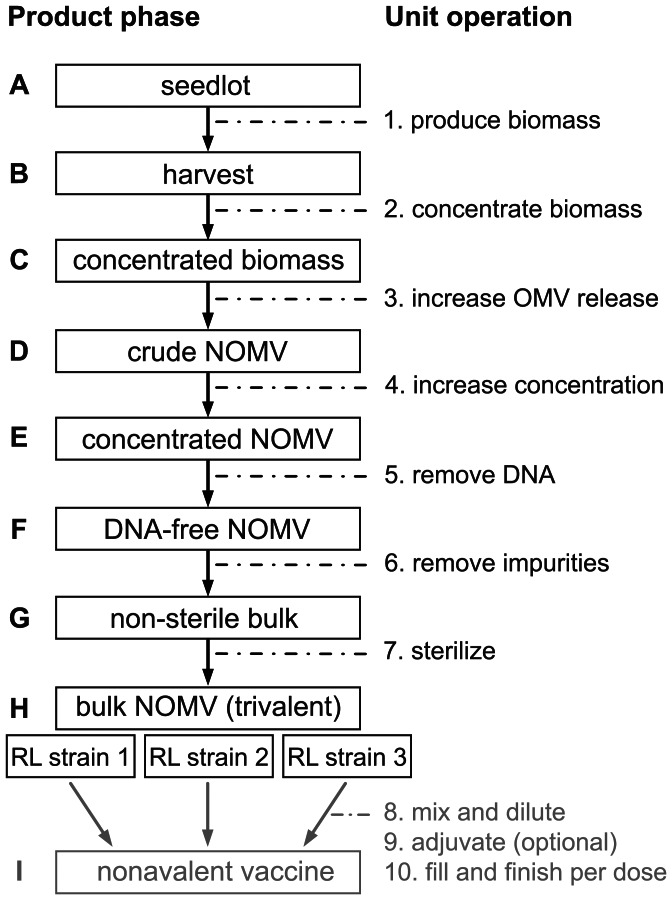
Flow-chart for improved NOMV production. Process performance is evaluated with the NonaMen concept, a nonavalent PorA vaccine comprised of three trivalent RL production strains (Δ*rmpM*–Δ*lpxL1* mutant strains; Supplementary Table S2). Production of trivalent bulk NOMV is depicted in phases A to H. The transition from one phase to the next requires a specific unit operation (1 to 10). The seedlot (A) is first expanded in shake flask and bioreactor pre-cultures, then used to inoculate the production bioreactor. Harvested cells (B) are concentrated with microfiltration (C), and vesicle release is stimulated with a detergent-free buffer containing the chelating agent EDTA (D). Cells are discarded, and the crude NOMV are concentrated with ultrafiltration (E). Any residual DNA is digested with nuclease (F), and the extract is purified with gel filtration chromatography. The non-sterile bulk (G) is then sterilized by filtration to obtain the bulk NOMV (H). The trivalent product can be stored for at least one year before mixing as nonavalent vaccine and diluting to dose concentration (I).

The improved NOMV production process is outlined in Figure 1. Product phases A to H describe the production of bulk NOMV. The upstream process starts with thawing of a working seedlot (phase A). After multiple passages of growth, the 40 L production culture is harvested (phase B), and biomass is concentrated with microfiltration (phase C). After a detergent-free treatment using the chelating agent EDTA (ethylenediaminetetraacetic acid), biomass is removed with consecutive centrifugation and filtration steps to obtain the pathogen-free, crude NOMV (phase D), which are the input material for downstream processing. Crude NOMV are concentrated and washed with ultra(dia)filtration (phase E), and DNA is digested with nuclease (phase F). The resulting NOMV are purified with gel filtration to remove salts and digested DNA fragments and also to enable a buffer change to storage buffer (phase G). After sterile filtration (phase H), the bulk NOMV from strains with trivalent PorA can be stored until they are combined as a nonavalent vaccine and diluted to the appropriate dose concentration (phase I). The bulk NOMV vaccines in this study were produced for use in preclinical evaluation. As a starting point for process development, specifications were defined for processing aspects based on analysis of three reference processes: one large-scale NOMV process [31] and two large-scale DOMV processes [20], [21]. Supplementary Table S1 compares these references with the current process. Table 1 summarizes the solutions designed to meet the specifications and their current status of implementation.

**Table 1:** Specifications for improved NOMV production.

unit operation	specification	solution/result	current status
1. produce biomass	reproducible growth	chemically defined growth medium	optimized
	scalable >500 L	40 L bioreactor, scalable to 800 L	optimized [34]
	no antifoam	low-shear foam breaker	optimized [34]
	on-line harvest point decision	oxygen consumption monitoring	optimized
2. concentrate biomass	replace centrifugation	hollow fibre microfiltration	functional [35]
3. increase OMV release	mild, low-shear	blender omitted	functional
	high extraction efficiency	critical process parameters identified	optimized [35]
	aseptic biomass removal	continuous centrifugation	not functional
	inactivation without phenol	depth filtration 0.5–0.2 µm	optimized
4. increase concentration	replace ultracentrifugation	ultrafiltration 100 kD cutoff	functional
5. remove DNA	replace ultracentrifugation	nuclease treatment	functional
6. remove impurities	replace ultracentrifugation	gel filtration chromatography	optimized
7. sterilize	replace thiomersal	sterile filtration 0.2 µm	optimized
	high efficiency	detergent-free (prevents aggregation)	optimized [35]

An outline with 7 consecutive unit operations (corresponding to Figure 1) was used as a starting point for process development and optimization. One large-scale NOMV process [31] and two large-scale DOMV processes [20], [21] were used as references to define desired specifications for each unit operation (Supplementary Table S1). Solutions that are expected to provide these specifications are summarized, together with their implementation status in the current production process.

### Bacterial strains

The three RL production strains (Δ*rmpM*–Δ*lpxL1* mutant strains; Supplementary Table S2) with different combinations of PorA antigens were adapted to the production medium and stored as frozen master and working seedlots. Quality Control testing confirmed microbiological identity (*N. meningitidis*), bacterial monoculture (no contaminating organisms), cell viability (>10^5^ cfu/mL), and PorA identity (trivalent). Seedlots were produced, released, and stored according to GMP guidelines and may therefore be used to produce vaccines for clinical evaluation. Genetic stability of the RL production strains was constant at all four time-points that were assessed: master seedlot (N = 0 generations of growth), production harvest (N = 29±1 generations), and two post-production time-points (N = 43±2 and N = 61±1 generations of growth, respectively). PorA identity results matched the subtypes in Supplementary Table S2, as did the LPS identity (*galE*-*lpxL1*-LPS). Gene disruptions of *rmpM* and *porB* were confirmed with PCR (data not shown). Disruption of the *cps* locus (*siaA*, *siaD* and *galE* genes) was confirmed at all time-points by testing functionality of the erythromycin resistance gene (*ery^R^*) used for disruption. Comparable tests confirmed kanamycin and chloramphenicol resistance at all time-points, providing supplementary confirmation of *lpxL1* and *rmpM* disruption. These results indicate that the RL production strains are genetically stable for at least 30 generations of growth beyond the usual production harvest, in the absence of selective antibiotics.

### Upstream production process

**Figure 2:**
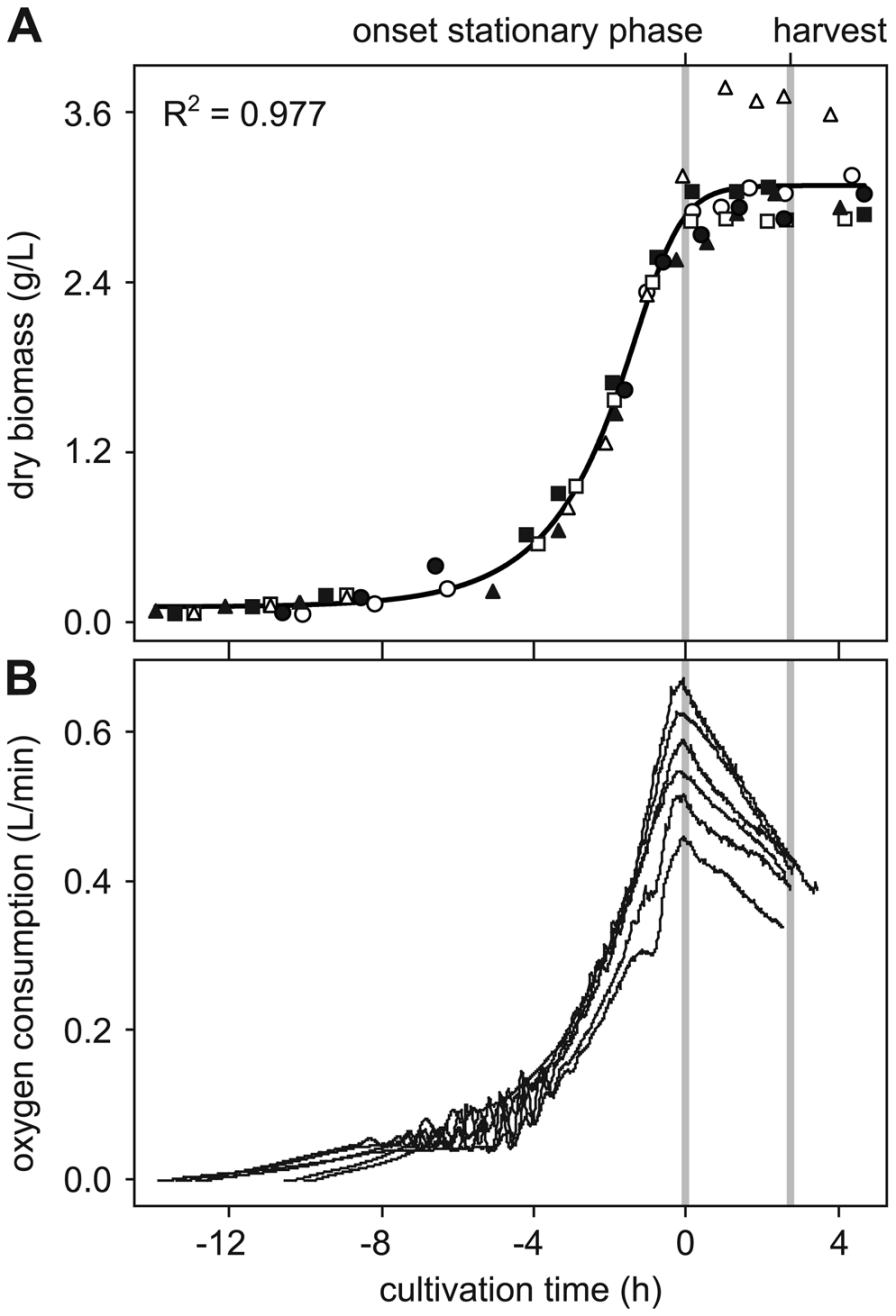
Upstream process characteristics. A) The upstream process is performed at 40 L scale, which is representative for large-scale (800 L [34]). Biomass growth (black line) on chemically defined production medium is highly reproducible (regression R^2^ = 0.977). Cultivations from RL production strain 1, 2, and 3 are indicated by squares, circles, and triangles (respectively). Duplicates for each strain are indicated with open and closed symbols, giving a total of 6 cultivations. B) Oxygen consumption is monitored continuously. Cultivations are aligned at the time-point of maximal oxygen consumption (t = 0), which represents onset of the stationary phase. Harvest point of the cultivations has previously been optimized at t = 3 hours [35].

The primary pre-culture was inoculated with a thawed working seedlot, grown in a shake-flask to an OD_590_ of 1.4±0.2 and used to inoculate the secondary pre-culture, which was grown in bioreactors to an OD_590_ of 2.9±0.9. Inoculants were transferred during the exponential growth phase. All cultures were grown on chemically defined growth medium, without antifoam, and a mechanical foam breaker was implemented for the production bioreactor [34]. Production cultures started at an OD_590_ of 0.12±0.02, at 40 L cultivation volume. Biomass growth was monitored by measuring OD_590_ and expressed as biomass concentration (1 OD_590_ unit corresponds to 0.32 g/L dry biomass or 2.47 g/L wet biomass). In addition, oxygen consumption was monitored on-line to determine the harvest point. The resulting characteristics are shown in Figure 2. Biomass growth was highly reproducible for all cultivations (n = 6; duplicates for each strain; R^2^ = 0.977). Cultivation profiles were aligned at the time-point of maximal oxygen consumption, which represented the onset of the stationary phase (t = 0). The production cultures were harvested for further processing at 3.1±0.3 hours after onset of the stationary phase, with a wet biomass yield of 23.8±2.2 g/L. Harvest was concentrated 5–8 fold with microfiltration (scalable, aseptic) and washed with 2.1±0.2 volumes Tris-HCl pH 8.6 buffer to adjust pH. This ensured a pH of 8.3–8.6. The EDTA treatment was performed with optimal setpoints for ‘harvest’ and ‘pH’, which were previously identified as critical process parameters [35].

### Downstream production process

**Figure 3:**
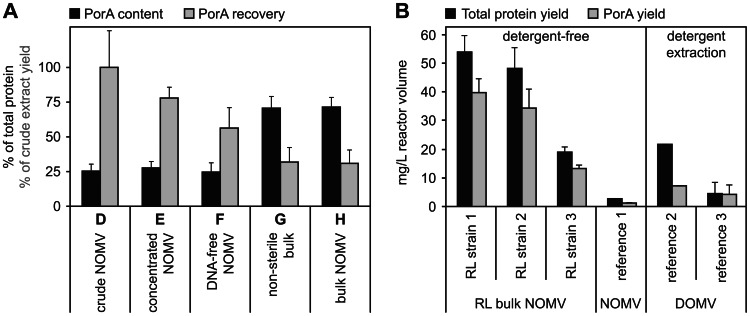
Downstream process characteristics. A) PorA content and recovery are shown for the consecutive product phases of the downstream process, from crude NOMV to bulk NOMV (Figure 1, phase D to H). PorA content (black bars) reaches final values from phase G onwards, indicating that the gel filtration chromatography effectively removes impurities. PorA recovery relative to phase D (grey bars) shows that losses are spread equally across the process, but notably not during sterile filtration (phase G to H), resulting in a reproducible and good overall recovery. Error bars indicate standard deviation of combined data from the three RL production strains (n = 9). B) Total protein and PorA yield of RL bulk NOMV (phase H) and large-scale reference processes. Reference 1 [31] is most comparable the current process since both are detergent-free (NOMV product). References 2 [20] and 3 [21] use detergent-extraction (DOMV product). All RL strains have a higher PorA yield than the references. RL strain 3 has a lower yield than the other two RL strains, resulting in a total protein yield that is comparable to reference 2 but still higher than the other references. The results of individual RL strains are reprodicible, therefore this yield difference is not caused by the process. Error bars indicate standard deviation of replicate batches (n = 3).

In the NonaMen vaccine concept, PorA is the primary protective antigen, and therefore a high PorA content is desired. When NOMV from the trivalent RL strains are properly purified, PorA content comprises more than 55% of total protein present in the vesicles [22], [35]. Figure 3A shows how PorA content evolves through the phases of the downstream process. During phases D, E and F, it was relatively low at an average of 26±5% of total protein. Gel filtration chromatography removed impurities effectively and reproducibly, resulting in a final PorA content of 71±7% at phases G and H. This was significantly higher than at the previous phases (p<0.0001). Figure 3A also shows the PorA recovery at various production phases. Initial PorA yield after EDTA treatment of cells (phase D) was set at 100%, and recovery at downstream phases (E to H) was expressed as percentages of the initial yield. Losses were distributed evenly across phases E, F and G (22±8%, 22±7%, and 25±7% yield loss, respectively), resulting in a good overall PorA recovery of 31±10%. PorA recovery for RL strain 3 was lower than for the other strains, resulting in slightly elevated standard deviations. Notably, sterile filtration (phase G to H) was performed with a recovery of 96±6%, indicating that the OMV passed the 0.2 µm filter without detectable losses.

### Process yield

Mean yield of the novel process was substantially higher than the yield of large-scale reference processes, as illustrated in Figure 3B. Reference 1 [31] is most comparable to the current process, since they are both detergent-free, whereas references 2 [20] and 3 [21] use detergent-extraction (Supplementary Table S1). The mean yield of three RL production strains was 40±17 mg total protein per L reactor volume, which was 15-fold higher than reference 1 (2.7 mg/L), 2-fold higher than reference 2 (21.7 mg/L), and 9-fold higher than reference 3 (1.9–7.4 mg/L). However, the individual RL strains varied: RL strain 3 had a yield of 19±2 mg/L, which was considerably less than RL strains 1 and 2 (54±6 and 48±7 mg/L, respectively). Figure 3B therefore shows the individual RL yields rather than their average. In addition, Figure 3B confirms a substantially higher PorA yield for all RL strains (2–30 fold). According to the literature, yield of reference 1 was based on 270 L reactor volume, 450 mL product with 0.8 mg/mL total protein and PorA content of 50% (our estimation); yield was multiplied by 2.03, because 49.3% (not 100%) of total biomass was processed. Reference 2 yield was based on 15 L reactor volume; 13000 doses (25 µg total protein with 33% PorA). Reference 3 yield was based on 135 L reactor volume; 250 to 1000 mg total protein with 89% PorA.

### Quality Control

The most important aspect of process performance is the resulting product quality, in this case the quality of bulk NOMV with trivalent PorA. Therefore, batches of bulk NOMV (n = 9; three for each RL production strain) were characterized with Quality Control tests according to GMP and European Pharmacopoeia (EP) guidelines. The analyses confirmed that quality was within preliminary specifications for all batches (Table 2A). The NOMV were sterile, had the expected PorA identity (trivalent), high total protein content (1.4±0.2 mg/mL), high total PorA content (0.98±0.09 mg/mL), and low residual DNA (0.007±0.002 mg/mL). They also had native amounts of *galE*-*lpxL1*-LPS (0.23±0.06 mg/mL), homogeneous OMV size (81±7 nm), and absence of aggregation (3±3%). Three batches of bulk NOMV (one for each trivalent RL strain) were assessed for endotoxic activity, functional immunogenicity, stability, and appearance (see paragraphs below). In addition, bulk NOMV were mixed to produce nonavalent vaccine, for evaluation in mice and rabbits. This separate study confimed that the nonavalent vaccine induces broad immunogenicity and is well-tolerated [36].

**Table 2:** Quality Control testing of bulk NOMV.

**2A – general characteristics**				
**QC parameter**	**preliminary specification**		**result (n = 9)**	
sterility	no growth		no growth	
PorA identity	trivalent		trivalent	
total protein	>1.0 mg/mL*		1.4±0.2 mg/mL	
total PorA	1.00±0.25 mg/mL*		0.98±0.09 mg/mL	
PorA content	>55% of total protein		71±7%	
*galE*-*lpxL1*-LPS	0.25±0.15 mg/mL		0.23±0.06 mg/mL	
OMV size	<220 nm		81±7 nm	
aggregation	no specification		3±3%	
DNA	<0.050 mg/mL		0.007±0.002 mg/mL	
**2B – endotoxic activity**				
**vaccine**	**preliminary specification**		**result (ng/mL IL-6 per human dose)**	
bulk NOMV (n = 3)	<DTP-IPV reference		27±2	
DOMV (low toxicity)	–		700	
DTP-IPV reference	–		778	
**2C – serum bactericidal activity**				
**vaccine**	**PorA subtype**	^2^log titre**		**responders**
bulk NOMV strain 1 (n = 1)	P1.7,16	10.3 (>4)		9/10
	P1.5-1,2-2	12.0 (>4)		10/10
	P1.19,15-1	4.6 (>4)		9/10
bulk NOMV strain 2 (n = 1)	P1.22,14	8.7 (>4)		10/10
	P1.7-1,1	6.2 (>4)		5/10
	P1.18-1,3,6	3.0 (<4)		1/10
bulk NOMV strain 3 (n = 1)	P1.5-2,10	10.2 (>4)		10/10
	P1.12-1,13	7.7 (>4)		10/10
	P1.7-2,4	6.4 (>4)		7/10

A) General characteristics include biochemical composition, PorA identity, and sterility for which preliminary specifications were set. B) Endotoxic activity of the bulk NOMV was approximately 25-fold lower than in DOMV and DTP-IPV reference vaccines. C) In addition, high bactericidal titres were induced in mice (^2^log titres >4 for 8 out of 9 PorA subtypes), with few non-responders. These results indicate that the bulk NOMV have a consistent product quality and are likely to be safe and immunogenic in humans. *During processing PorA concentration of bulk NOMV is set at 1 mg/mL for storage purposes. **Mean titre of responders.

The endotoxic activity of bulk NOMV (n = 3) was measured with IL-6 monocyte activation (Table 2B). Used as references were a DOMV vaccine with low toxicity, as proven in a phase I clinical study [37], and a DTP-IPV vaccine (containing diphtheria and tetanus toxoid, whole-cell pertussis, and inactivated polio virus). The bulk NOMV induced 27±2 ng/mL of IL-6 per human dose (26, 27 and 29 ng/mL for RL strains 1, 2 and 3, respectively; one human dose corresponded to 15 µg PorA of each PorA subtype). This was approximately 25-fold lower than both reference vaccines. The DOMV vaccine induced 700 ng/mL of IL-6 per human dose, and the DTP-IPV vaccine induced 778 ng/mL of IL-6 per human dose. Therefore the NOMV are expected to be safe for parenteral use in humans.

Functional immunogenicity was assessed by measuring serum bactericidal activity in mice sera after two immunizations with trivalent bulk NOMV, one for each RL strain (Table 2C). High bactericidal titres (>4 fold increase in mean ^2^log titre of responders) were observed for 8 of 9 tested PorA subtypes. PorA subtype P1.18-1,3,6 had a low titre, with only 1 mouse of 10 responding to vaccination. Of the 8 PorA subtypes with high titres, P1.19,15-1 was just above threshold, and P1.7-1,1 had a lower number of responders (5 out of 10 mice). These results indicate that the bulk NOMV induced high functional immunogenicity in mice for most PorA antigens.

**Figure 4:**
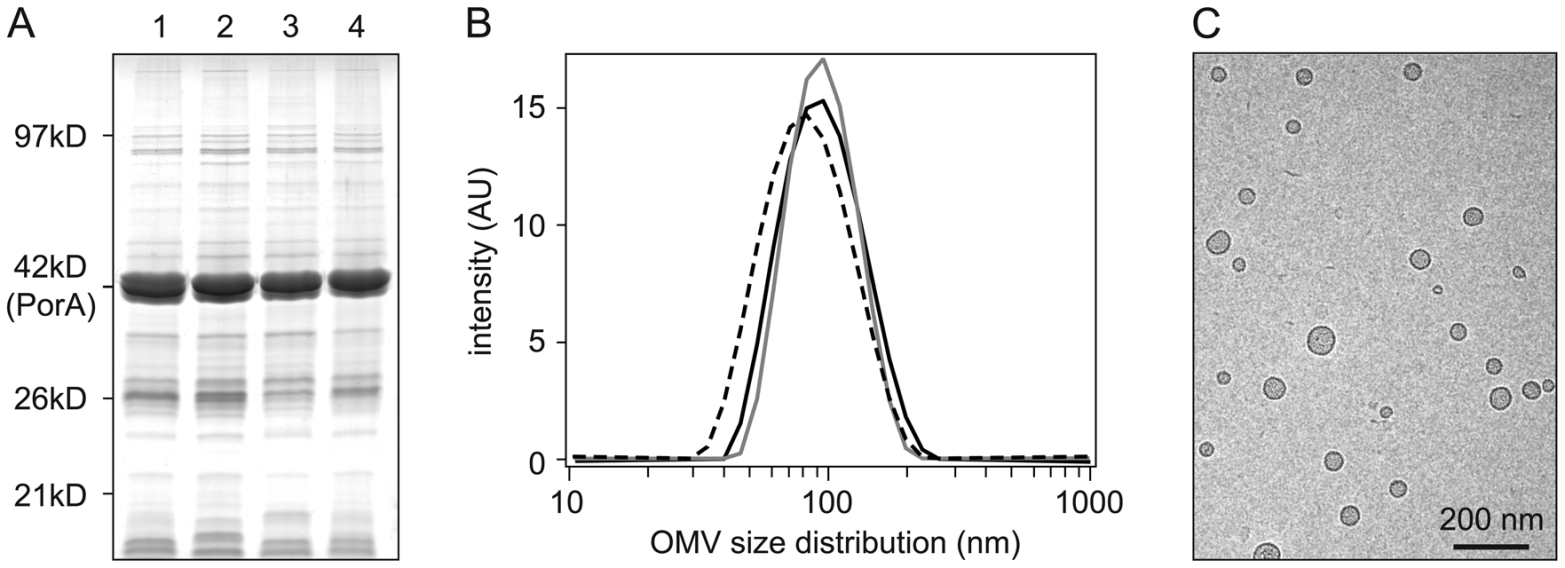
Protein composition and appearance of NOMV. A) Protein composition of trivalent bulk NOMV is shown in lanes 1 to 3 for RL strains 1, 2 and 3, respectively (phase H), and composition of the nonavalent vaccine (phase I) in lane 4. Bulk NOMV from the three RL production strains are comparable, therefore the nonavalent vaccine has an equally comparable protein composition. B) Vesicle size of trivalent bulk NOMV is similar for RL strain 1 (black line), RL strain 2 (grey line), and RL strain 3 (dashed line). All bulk NOMV are non-aggregated and have comparable, homogeneous size distributions, with averages between 74 and 87 nm. C) Electron micrograph of nonavalent vaccine (non-adjuvated) after dilution to dose concentration shows that vaccine contains fully intact, non-aggregated OMV, an important benefit of the detergent-free production process.

In addition to the Quality Control tests, we assessed protein composition and appearance of the NOMV. Total protein composition (determined by SDS gel electrophoresis) is shown in Figure 4A, illustrating the major contribution of PorA protein (42 kD; 71±7% of total protein). Overall protein composition of bulk NOMV was comparable among the three trivalent RL production strains, resulting in nonavalent vaccine with an equally comparable composition. Dynamic light scattering (DLS) was used to visualize NOMV appearance (Figure 4B) and found non-aggregation; homogeneous size distribution with averages of 86.2 nm (RL strain 1), 86.7 nm (RL strain 2), and 73.8 nm (RL strain 3); and low polydispersity indices of 0.12, 0.08, and 0.11, respectively. A representative electron micrograph of non-adjuvated nonavalent vaccine after dilution to dose concentration (Figure 4C) showed fully intact, non-aggregated vesicles with a size between 40 and 100 nm.

### NOMV stability

The production dates of three batches of bulk NOMV (one for each RL strain) were used as starting points for an ongoing real-time stability study. The bulk NOMV were stored at a total PorA concentration of 1 mg/mL, at 4°C in the dark. Quality Control tests focused on biochemical aspects (PorA content, OMV size, aggregation, and pH), sterility, and epitope concentration (determined by quantitative Biacore assay with specific antibodies against a PorA subtype, using one representative PorA selected for each RL strain). Stability data at 0, 3, 6 and 12 months after production are presented in Supplementary Table S3. The data did not contain trends with significant non-zero slopes, indicating that the NOMV were sterile and had a stable PorA content, vesicle size, and pH. Aggregation was low at all time points (<5%), but trend analysis could not be performed due to skewed distribution. Epitope concentrations seemed constant, but sample size at t = 12 was still too small for reliable trend analysis. The table shows results for all time-points up to t = 12, indicating that the bulk NOMV were stable for at least one year after production. Trivalent bulk NOMV were also mixed to produce nonavalent vaccine [36]. Functional immunogenicity (SBA) of this vaccine in mice will be monitored up to 36 months after production in a second, real-time stability study (data not yet available).

## Discussion

An efficient and consistent process for the detergent-free production of NOMV vaccine against *N. meningitidis* (Figure 1) has been developed. Compared to previously described processes, the new process offers important improvements that are likely to enable production at commercial scale (Table 1; Supplementary Table S1) [20], [21], [31]. Process performance was evaluated with the NonaMen vaccine concept, which provides broad protection against serogroup B meningococcal disease based on nine different PorA antigens [10], [11], [38].

The upstream process uses production strains with multiple recombinant antigens that are genetically stable in the absence of selective antibiotics. The strains also have mutations in *lpxL1* and *rmpM* genes to attenuate LPS toxicity and improve yield [22], [23] (Supplementary Table S2). Without these genetic modifications, the detergent-free approach would not be feasible. To prevent hydrophobic interactions with the outer membrane, antifoam is omitted from the growth medium. Instead, a mechanical foam breaker was used that does not impose significant shear force or lysis [34]. The resulting growth curves are highly reproducible (Figure 2A) and with a higher biomass yield than large-scale references [20], [31]. Biomass yield can be further improved by implementing a feed strategy, since onset of the stationary phase is triggered by nutrient depletion rather than limitations in the oxygen supply (data not shown). The production culture is harvested after three hours of the stationary phase, and the harvest point is detected online with oxygen consumption monitoring (Figure 2B). Biomass density is constant during this period, but the oxygen consumption does not stabilize. It is therefore uncertain whether the stationary phase is a temporary transition from exponential growth to death phase, or a truly stationary phase in which metabolism remains at maintenance levels for a prolonged period.

Harvested biomass is concentrated with scalable, aseptic equipment, and NOMV release is stimulated with a detergent-free EDTA buffer under optimized conditions [35]. The EDTA treatment does not require a blender, which is likely to increase lysis. Spent biomass is then separated from the crude NOMV with consecutive centrifugation and depth-filtration. These steps remove all pathogenic activity from the crude NOMV without the need for hazardous chemicals like phenol. However, the centrifugation still requires handling in a biohazard cabinet, with risk of contamination, and limits the process scale to approximately 100 L cultivation volume. Therefore future improvements will include the implementation of alternatives, like continuous flow centrifugation for initial biomass removal, as demonstrated for *Bordetella pertussis* and fragile mammalian cells [39], [40].

During downstream processing (Figure 3A), the crude NOMV are concentrated with ultrafiltration, and nuclease is added for digestion of any DNA. Subsequent gel filtration chromatography removes impurities and ensures a high final PorA content. These steps replace ultracentrifugation, which is commonly used and effective but limits the production scale. Ultracentifugation also requires manual homogenization of multiple NOMV pellets. This is performed in a flow cabinet, with risk of contamination, and followed by a microfluidizer treatment that imposes excessive shear force. The current process uses scalable, aseptic equipment and allows the NOMV to remain a stable colloid suspension during all production stages. Overall PorA recovery is good, approximately one third of the initial crude NOMV yield. The ultrafiltration and nuclease steps give reproducible results but are not yet optimized, resulting in higher losses and offering room for future improvement. Importantly, sterile filtration of bulk NOMV is performed without detectable losses and obviates the need for preservatives like thiomersal. This is an notable benefit of the detergent-free approach, ensuring that the NOMV are non-aggregated and smaller than the filter cut-off (<220 nm). Detergent-extracted OMV are prone to aggregation and more heterogeneous in size, resulting in significant losses during sterile filtration [21], [22], [41]. Even though these issues do not affect immunogenicity, it is clearly beneficial to prevent them by omitting the detergent-extraction [42].

Overall PorA yield of the new production process is substantially higher than the yield of published reference processes with or without detergent-extraction (Figure 3B) [20], [21], [31]. This is a cumulative benefit of production strains with *rmpM* mutation [22], a higher biomass yield, and a generally more efficient process. RL strain 3 had a lower yield than the other strains but not as a result of the process, which gave reproducible results for all three strains. The lower yield may originate from a less effective assembly of recombinant PorA antigens in strain 3, but this remains speculative. One of the reference processes [20] was commercialized to control a meningitis epidemic in New Zealand. Compared to that reference, the current process shows a higher PorA yield for all RL strains, avoids components of animal origin (deoxycholate), and is scalable to at least 800 L cultivation volume [34]. Therefore it is likely to be feasible at commercial scale.

Quality Control testing showed that product quality is within preset criteria for all 9 bulk NOMV batches, an important indicator for process performance (Table 2A). The product quality has been constant for at least the first year of an ongoing real-time stability study (Supplementary Table S3). This study seeks to show stabililty for up to three years. Endotoxic activity of the bulk NOMV is approximately 25-fold lower than a low-toxicity vaccine based on detergent-extracted OMV [37] or the DTP-IPV vaccine with whole-cell pertussis component. Long used in the Dutch national vaccination program, that DTP-IPV vaccine had a relatively high rate of adverse events and was replaced in 2005 with one containing acellular pertussis [43], [44]. It thus represents the upper range of low endotoxic activity, providing a useful context for our results. Functional immunogenicity of the trivalent bulk NOMV in mice is high, and comparable to detergent-extracted HexaMen and NonaMen vaccine [10], [21]. The improved production process therefore generates high quality NOMV that are likely to be well-tolerated and induce a functional immune response in humans (Table 2B-C). The contribution of individual PorA's needs to be assessed in more detail, since subtypes P1.7-1,1 and P1.18-1,3,6 gave low titres or few responders. Other studies with multivalent PorA vaccine confirm this observation [10], [36]. Priming with monovalent OMV from less immunogenic PorA subtypes before immunization with the multivalent vaccine may solve this issue [45]. A recent study with mice and rabbits confirmed that NonaMen vaccine from the improved process induces broad immunogenicity without related toxicity or pathology [36]. These results encourage clinical evaluation.

The scope of the novel production process is not restricted to the NonaMen concept and may be applicable for the late-stage development of other NOMV vaccines. These include promising serogroup B concepts with a different antigen composition or vaccines against other *N. meningitidis* serogroups [12], [13], [29], [30], [32], [33], [46]. The outer membrane vesicles from gram-negative pathogens in general, like *Escherichia coli*, *Vibrio cholera*, or *Bordetella pertussis*, are also within the scope if the upstream process is adjusted to meet the nutritional requirements of these pathogens [47], [48], [49], [50], [51], [52], [53]. Even recombinant antigens derived from other pathogens may benefit from an OMV presentation form [24], [54], [55], [56]. This work thus provides a robust production platform to facilitate the late-stage development and clinical evaluation of NOMV vaccines.

## Materials and Methods

### Ethics statement

Animal experiments were performed at the Animal Care Facility of the National Institute for Public Health and the Environment (RIVM, The Netherlands), according to current animal welfare regulations. The institutional Animal Ethical Review Committee (DEC) of the National Institute for Public Health and the Environment evaluated and approved the experiments according to Dutch legislation (application number DPA2011-00014). All efforts were made to minimize animal suffering.

### Trivalent strains and seedlots

The NOMV vaccines in this study were produced with RL strains 1, 2 and 3 (Supplementary Table S2). Deletion of *rmpM* (R) and *lpxL1* (L) genes improved yield and attenuated LPS toxicity [22]. The RL strains are non-encapsulated variants of the *N. meningitidis* serogroup B isolate H44/76 [57] derived by deletion of the *cps*-locus (*siaA*, *siaD* and *galE* genes). Each RL production strain has 3 cloning sites for recombinant antigens, which were used to express unique PorA subtype variants (NonaMen concept; nonavalent PorA). For each strain, the research seedlot was adapted to the production medium in Erlemeyer shake flasks to obtain frozen master seedlots (Figure 1, phase A). One master seedlot was used to generate approximately 50 frozen working seedlots through two additional expansions in shake flasks (two-tiered seedlot system). Master and working seedlots (cells at OD_590_ = 1.5±0.3; stored at −135°C with 17% (v/v) glycerol) were produced according to GMP guidelines.

### Production of NOMV

Bulk NOMV were produced according to standard operating procedures in a non-GMP pilot facility, but future use of the process in a GMP facility was anticipated. Evaluation of deviations, batch review, and batch release were performed by QA/QP officers according to GMP standards. All cultures were grown in chemically defined production medium free of animal components and containing glucose, amino acids, salts, iron, and trace elements [34]. A primary 150 mL pre-culture was inoculated with 10 mL working seedlot and incubated in disposable 500 mL shake flasks with vented closure (Nalgene, Rochester NY, U.S.A.) at 35°C, 200 rpm. The pre-culture was used to inoculate a secondary pre-culture, grown in a 5 L bioreactor with 3 L working volume (Applikon, Schiedam, The Netherlands). At OD_590_ values between 1.5 and 4.5, a fixed amount of bacteria (corresponding to 1 L at OD_590_ = 3) was transferred to the 60 L production bioreactor with 40 L working volume (Applikon, Schiedam, The Netherlands). Bioreactors were operated as described previously [34]. Antifoam was omitted, and a mechanical foam breaker was used to control foaming at 40 L scale (the 5 L reactor did not require a foam breaker) [34]. The production culture was harvested 3 hours after onset of the stationary phase (Figure 1, phase B), as observed with online monitoring of the oxygen consumption. Biomass was concentrated 5-fold using hollow-fiber microfiltration units with 0.2 µm pore size and 1.8 m^2^ surface area (Spectrum Laboratories, Rancho Dominguez CA, U.S.A). Circulation was constant at 6.5 L/min, and feed pressure was monitored (phase C). Concentrated biomass was then diafiltrated with the microfiltration unit, using 2 volumes of buffer (100 mM Tris-HCl, pH 8.6). Concentrated EDTA solution was added to a final concentration of 10 mM to stimulate NOMV release (30 min at ambient temperature with continuous stirring). Bacteria were separated from the crude OMV by centrifugation (6 buckets with a content of max. 1 L; 75 min., 12500×g, 4°C), and the supernatant was depth-filtered (0.5–0.2 µm) to remove any residual pathogens (phase D). In phase E, the crude OMV extract was concentrated 12-fold and washed with ultra(dia)filtration (100 kD cut-off; 100 mM Tris-HCl pH 8.6). In phase F, any DNA that might be present was digested with 1000 U/L Benzonase (Merck; Schiphol-Rijk; The Netherlands). To remove impurities (bacterial host proteins, salts, small DNA), the NOMV were loaded onto a gel filtration chromatography column packed with Sepharose 6 Fast Flow size exclusion matrix (GE Healthcare, Hoevelaken, The Netherlands). During gel filtration (phase G), the buffer was changed to storage buffer containing 10 mM Tris-HCl pH 7.4 with 3% (w/v) sucrose [42]. Bulk NOMV were sterilized with filtration (0.22 µm; Pall, Mijdrecht, The Netherlands), diluted to storage concentration (1 mg total PorA/mL), and safely stored in the dark at 2–8°C until further use (phase H). Stability of the bulk NOMV under these conditions is at least one year (ongoing real-time stability study). To prepare nonavalent vaccines for preclinical evaluation purposes (phase I), aseptic fill and finish were performed according to GMP guidelines. First, bulk NOMV from three trivalent RL strains were mixed to achieve nonavalent vaccine, then diluted to dose concentration with storage buffer, based on PorA concentration. Vials with with 0.6 mL extractable volume were filled with vaccine and labeled. Each vaccine dose (0.5 mL) contained 15 µg of each PorA with a total of nine different PorA subtypes (Supplementary Table S2).

### Quality Control

QC tests were performed according to GMP and European Pharmacopoeia guidelines. **PorA identity** was verified with qualitative ELISA, using specific antibodies against each PorA subtype [21]. OMV from monovalent PorA strains and a Δ*porA* strain were used as controls**. Total protein concentration** (Ph. Eur. 2.5.33) was measured with the Lowry protein assay. Peterson's modification was used to reduce the effect of interfering substances [58]. The assay was performed according to manufacturer's protocol (Sigma-Aldrich, Zwijndrecht, the Netherlands). PorA content was determined by SDS gel electrophoresis (Ph. Eur. 2.2.31) followed by total protein staining and quantification of the 40–44 kD PorA bands. Gels were stained with Novex Colloidal Blue (Invitrogen, Breda, The Netherlands), and PorA was quantified as a percentage of total protein using TL100 1D gel analysis software (Totallab, Newcastle upon Tyne, U.K.) [58], [59]. Total nucleic acid concentration was used to estimate **residual genomic DNA**. Samples were incubated with ethidium bromide solution (MP Biochemicals, Illkirch, France), and fluorescence intensity was measured to quantify DNA concentration based on a salmon sperm DNA standard (Invitrogen, Breda, The Netherlands) [34], [60]. For bulk NOMV and nonavalent vaccines, there was no need to discriminate between RNA and DNA with an RNAse pre-treatment, because total nucleic acid concentration was low (<0.06 mg/mL). RNAse pre-treatment was used for in-process controls on intermediate products (before phase F). Fatty acid composition was analyzed to quantify **LPS concentration** with a modified gas chromatography method [61], [62] (Ph. Eur. 2.2.28). LPS was isolated with hot phenol-water extraction [63] and quantified using the peak height of C14:0-3OH, with C12:0-2OH as the internal standard (two C14:0-3OH residues per LPS). The molecular weight of *galE*-*lpxL1*-LPS in the RL strains was previously estimated at 2848 g/mol [22], but recent mass spectrometry work provided a more reliable estimation of 3191 g/mol, which was used for this study (Pupo Escalona *et al.*, manuscript in preparation). **Identity of** ***galE*** **-** ***lpxL1*** **-LPS** was verified with mass spectrometry [64]. An aliquot of 200 µL isolated LPS (see above; 50 nmol/ml) was freeze-dried and taken up in 0.1 ml of 2% acetic acid (pH 2.8). The mixture was heated to hydrolyze the LPS and release the lipid A moiety. Chloroform-methanol (2∶1 v/v) was used to extract the lipid A and, after phase separation, the upper phase was used for analysis with mass spectroscopy (nano electrospray tandem MS on a Finnigan LCQ instrument in the negative-ion mode) [65]. **Vesicle size distribution** was measured with dynamic light scattering (DLS) at 25°C with a Malvern 4700 system [22] (Ph. Eur. 2.9.31). The vesicle size distribution was reflected in the polydispersity index (PdI), which ranges between 0.0 (fully homogeneous size distribution) and 1.0 (random size distribution). **Aggregation** was quantified by comparing the protein content of the NOMV starting material with the protein content of the supernatant after centrifugation at low speed (10 min. at 5000 xg) [22], using the total protein assay described above. Aggregation was calculated by expressing the difference between the total protein content of supernatant and starting material as a percentage of the starting material. **Sterility** was determined by filtration (Ph. Eur. 2.6.1). Aliquots of 30 mL were filtered through a membrane (<0.45 µm pore size) to retain any contaminating organisms and remove constituents that might inhibit growth. The membrane was incubated in shake flasks with 180 mL tryptic soy broth (TSB) for 14 days at 30–35°C, with continuous aeration. The shake flask was inspected for visible growth and, if negative or sterile, it was inoculated with 10–100 cfu of *B. subtilis* and re-incubated as a positive control for medium quality**. Endotoxic activity** of bulk NOMV was measured as described previously by stimulating IL-6 production in human macrophage cell line MM6 [66] (Ph. Eur. 2.6.30) [22]. Macrophages were seeded in 96-well plates (3.75×10^4^ cells/well) in 125 µL IMDM medium supplemented with penicillin, streptomycin, L-glutamine, and fetal calf serum (Invitrogen, Breda, The Netherlands). Two-fold dilution series were made for all bulk NOMV samples in the supplemented IMDM medium, and cells were stimulated by adding 125 µL to each well. DOMV vaccine with known low toxicity (containing reduced amounts of non-attenuated *galE*-LPS) [37] and DTP-IPV vaccine (RIVM Bilthoven, The Netherlands) were included as references. A human dose of DTP-IPV vaccine contained 4 IU whole-cell pertussis, 30 IU diphtheria toxoid, 60 IU tetanus toxoid, and 40, 4 and 7.5 D-antigen units of inactivated poliovirus (type 1, 2 and 3, respectively). A human dose of OMV vaccine corresponded to 15 µg of each PorA subtype. Cells were stimulated for 16–18 hours at 37°C with 5% CO_2_, and IL-6 was quantified in the supernatant using ELISA, according to manufacturer's protocol (PeliKine Compact, Amsterdam, The Netherlands). Endotoxic activity was expressed in ng/mL IL-6 per human dose. **Serum bactericidal activity** of trivalent bulk NOMV was measured in sera of NIH mice (19–25 grams; <8 weeks old) after two subcutaneous immunizations on day 0 and 14 with 22.5 µg PorA (7.5 µg of each PorA subtype), with 10 mice per group. Sera were collected at day 28 and stored at −20°C. Bactericidal titres were measured as described before [67]. **PorA epitope concentrations** were quantified in bulk NOMV using biosensor analysis on a Biacore T100 (GE Healthcare Benelux, Diegem, Belgium). One representative PorA subtype was chosen for each RL production strain: P1.7,16 for strain 1; P1.22,14 for strain 2, and P1.12,13 for strain 3. Quantification was based on titration of free antibody after incubation with bulk NOMV. Recombinant PorA protein of a specific subtype was diluted in the presence of 0.05% (w/v) Zwittergent 3.14 and subsequently coupled to a CM5 sensor chip using an amine coupling kit according to manufacturer's protocol (GE Healthcare), with immobilization levels between 10.000 and 12.000 response units (RU). Dilution series of bulk NOMV in HBS-P buffer (GE Healthcare) were made, and samples were incubated overnight with PorA-specific monoclonal antibody in a suitable dilution. An OMV reference with known epitope concentration was also included. After incubation, the mixtures were centrifuged (5 min. at 3000×g) to remove any aggregates. The antibody excess was quantified by injecting samples on the sensor chip (10 µl/min for two minutes). Assay data were analyzed by fitting a four-parameter logistic curve with Biacore T100 evaluation software. PorA epitope concentrations of bulk NOMV were calculated relative to the reference OMV.

### Strain stability

Genetic stability of the RL production strains was assessed by continuing bacterial growth several generations beyond the normal harvest point of the production culture, using shake flasks. The age of the master seedlot was set at N = 0 generations, and experimental seedlots were stored at N = 30 generations of growth (regular production harvest), N = 45 generations (post-production A), and N = 60 generations (post-production B). The total number of generations at each time-point was calculated by taking the cumulative result of all previous passages in shake flasks and bioreactors. The number of generations from an individual passage was calculated with the formula: ln(ΔOD_590_)/ln(2), in which ΔOD_590_ was the difference between the initial and final OD_590_ of a passage. At each time-point, relevant genetic modifications of the RL production strains (Supplementary Table S2) were verified: PorA identity, identity of *galE*-*lpxL1*-LPS, *rmpM* and *porB* gene disruptions. Resistance against erythromycin, kanamycin, and chloramphenicol was tested by plating on GC agar with these antibiotics.

### NOMV stability

Stability of bulk NOMV was monitored in an ongoing three-year stability study. The results up to 12 months after production are currently available. Bulk NOMV (n = 3; one bulk product from each RL strain) were stored at a total PorA concentration of 1 mg/mL, at 4°C in the dark. At t = 0, 3, 6 and 12 months, PorA epitope concentration (one representative epitope for each bulk product), PorA content, OMV size, aggregation, and pH were tested as described above. Sterility was tested at t = 0 and t = 12 months after production. Results were assessed with trend analysis. Data from replicates and time-points were merged for each stability parameter and analyzed for normality and sufficient sample size (D′Agostino's K^2^ test; prerequisite for further analysis). A trend line was fitted with linear regression, and trends were tested for significant deviations from a non-zero slope (F test).

## Supporting Information

Table S1 **Comparison of large-scale OMV production processes.** The table shows how important OMV processing aspects are addressed in the current production process and in large-scale reference processes. Reference 1 [31] is most comparable to the current process, since both are detergent-free. References 2 [20] and 3 [21] use detergent-extraction. *The 40 L bioreactor has been successfully scaled to 800 L [34]. **Will be replaced with continuous centrifugation if scale-up to >100 L cultivation volume is required.
(PDF)

Table S2 **Genetic modifications of the trivalent RL production strains.** The RL strains are non-encapsulated variants of strain H44/76, in which *rmpM* (R) and *lpxL1* (L) genes have been disrupted to improve yield and attenuate LPS toxicity (*galE* truncated *lpxL1*-LPS). Each RL strain has 3 cloning sites for recombinant antigens. The NonaMen vaccine concept (nonavalent PorA) is used to evaluate performance of the new production process. Therefore all cloning sites of the trivalent RL strains contain PorA subtype variants. The RL strains are genetically stable for at least 30 generations of growth beyond the regular production harvest, in the absence of selective antibiotics.
(PDF)

Table S3 **Real-time stability of bulk NOMV.** Results of an ongoing stability study are shown at 0, 3, 6 and 12 months after production. They are presented as A) Quality Control tests for general stability aspects and B) Stability of selected PorA epitopes. Results are highly reproducible throughout the study, as confirmed by trend analysis, indicating that the bulk NOMV are stable for at least one year after production. *P-value >0.05 indicates that the time trend does not deviate significantly from a non-zero slope. **Skewed distribution and/or sample size too small. ***Missing data point.
(PDF)
