# Supplementary material for: Improved Production Process for Native Outer Membrane Vesicle Vaccine against Neisseria meningitidis
Source: PLoS One. 2013 May 31;8(5):e65157. doi: 10.1371/journal.pone.0065157 (PMC3669287; doi:10.1371/journal.pone.0065157)
Supplement: Table S1 — Comparison of large-scale OMV production processes. The table shows how important OMV processing aspects are addressed in the current production process and in large-scale reference processes. Reference 1 [31] is most comparable to the current process, since both are detergent-free. References 2 [20] and 3 [21] use detergent-extraction. *The 40 L bioreactor has been successfully scaled to 800 L [34]. **Will be replaced with continuous centrifugation if scale-up to >100 L cultivation volume is required. (PDF) [file pone.0065157.s001.pdf]

Supplementary Table S1

process aspect	current process	reference process 1	reference process 2	reference process 3
reduce LPS toxicity	Δ*lpxL1*	Δ*lpxL1*	deoxycholate	deoxycholate
components of animal origin	none	casamino acids	casamino acids, deoxycholate	deoxycholate
working volume bioreactor	40 L*	270 L	15 L	135 L
undefined medium components	none	casamino acids	casamino acids, yeast extract	yeast extract
reduce foam	foam breaker	antifoam	not described	not described
concentrate biomass	microfiltration	continuous centrifugation	centrifugation	cont. centrifugation, centrifugation
increase OMV release	Δ*rmpM*, detergent-free extraction	blender, detergent-free extraction	deoxycholate extraction	deoxycholate extraction
Inactivate biomass	depth filtration	phenol, heat	deoxycholate	deoxycholate
remove biomass	centrifugation**	centrifugation	centrifugation	centrifugation
increase OMV concentration	ultrafiltration	ultrafiltration	ultracentrifugation	ultracentrifugation
remove impurities	nuclease, gel filtration	nuclease, ultracentrifugation	ultracentrifugation	ultracentrifugation
sterilize	filtration	microfluidizer, filtration	thiomersal	thiomersal
