# Supplementary material for: Improved Production Process for Native Outer Membrane Vesicle Vaccine against Neisseria meningitidis
Source: PLoS One. 2013 May 31;8(5):e65157. doi: 10.1371/journal.pone.0065157 (PMC3669287; doi:10.1371/journal.pone.0065157)
Supplement: Table S2 — Genetic modifications of the trivalent RL production strains. The RL strains are non-encapsulated variants of strain H44/76, in which rmpM (R) and lpxL1 (L) genes have been disrupted to improve yield and attenuate LPS toxicity (galE truncated lpxL1-LPS). Each RL strain has 3 cloning sites for recombinant antigens. The NonaMen vaccine concept (nonavalent PorA) is used to evaluate performance of the new production process. Therefore all cloning sites of the trivalent RL strains contain PorA subtype variants. The RL strains are genetically stable for at least 30 generations of growth beyond the regular production harvest, in the absence of selective antibiotics. (PDF) [file pone.0065157.s002.pdf]

Supplementary Table S2

strain	cloning site 1	cloning site 2	cloning site 3	LPS type	other mutations	genetic stability
RL strain 1	*porA* P1.7,16	*porA* P1.5-1,2-2	*porA* P1.19,15-1	*ΔgalE*<br>*ΔlpxL1*	*Δcps*<br>*ΔrmpM*<br>*ΔporB*<br>ery^R<br>kan^R<br>cam^R	confirmed
RL strain 2	*porA* P1.22,14	*porA* P1.7-1,1	*porA* P1.18-1,3,6			confirmed
RL strain 3	*porA* P1.5-2,10	*porA* P12-1,13	*porA* P1.7-2,4			confirmed
