# Supplementary material for: Improved Production Process for Native Outer Membrane Vesicle Vaccine against Neisseria meningitidis
Source: PLoS One. 2013 May 31;8(5):e65157. doi: 10.1371/journal.pone.0065157 (PMC3669287; doi:10.1371/journal.pone.0065157)
Supplement: Table S3 — Real-time stability of bulk NOMV. Results of an ongoing stability study are shown at 0, 3, 6 and 12 months after production. They are presented as A) Quality Control tests for general stability aspects and B) Stability of selected PorA epitopes. Results are highly reproducible throughout the study, as confirmed by trend analysis, indicating that the bulk NOMV are stable for at least one year after production. *P-value >0.05 indicates that the time trend does not deviate significantly from a non-zero slope. **Skewed distribution and/or sample size too small. ***Missing data point. (PDF) [file pone.0065157.s003.pdf]

Supplementary Table S3

3A – general characteristics (n=3)						
**QC parameter**	**unit**	**t = 0**	**t = 3**	**t = 6**	**t = 12**	**trend p-value***
sterility	–	sterile	not available	not available	sterile	not applicable
PorA content	% total protein	72 ± 8	77 ± 3	72 ± 4	73 ± 5	>0.05 (0.91)
OMV size	nm	82 ± 7	82 ± 7	82 ± 7	81 ± 7	>0.05 (0.86)
aggregation	%	3 ± 4	1 ± 2	2 ± 2	0 ± 1	not available**
pH	–	7.4 ± 0.1	7.3 ± 0.2	7.4 ± 0.1	7.3 ± 0.2	>0.05 (0.61)

3B – epitope concentration (n=1)						
**bulk NOMV**	**unit**	**t = 0**	**t = 3**	**t = 6**	**t = 12**	**trend p-value***
RL strain 1 (P1.7,16)	mg/mL	0.39	0.41	0.53	0.55	not available**
RL strain 2 (P1.22,14)	mg/mL	0.50	0.47	NA***	0.47	not available**
RL strain 3 (P1.12,13)	mg/mL	0.63	0.54	0.57	0.50	not available**
